# Targeting of *Mycobacterium tuberculosis* Heparin-Binding Hemagglutinin to Mitochondria in Macrophages

**DOI:** 10.1371/journal.ppat.1002435

**Published:** 2011-12-08

**Authors:** Hosung Sohn, Jong-Seok Kim, Sung Jae Shin, Kwangwook Kim, Choul-Jae Won, Woo Sik Kim, Ki-Nam Min, Han-Gyu Choi, Je Chul Lee, Jeong-Kyu Park, Hwa-Jung Kim

**Affiliations:** 1 Department of Microbiology, Research Institute for Medical Sciences, College of Medicine, Chungnam National University, Daejeon, South Korea; 2 Department of Microbiology, Kyungpook National University School of Medicine, Daegu, South Korea; University of New Mexico, United States of America

## Abstract

*Mycobacterium tuberculosis* heparin-binding hemagglutinin (HBHA), a virulence factor involved in extrapulmonary dissemination and a strong diagnostic antigen against tuberculosis, is both surface-associated and secreted. The role of HBHA in macrophages during *M. tuberculosis* infection, however, is less well known. Here, we show that recombinant HBHA produced by *Mycobacterium smegmatis* effectively induces apoptosis in murine macrophages. DNA fragmentation, nuclear condensation, caspase activation, and poly (ADP-ribose) polymerase cleavage were observed in apoptotic macrophages treated with HBHA. Enhanced reactive oxygen species (ROS) production and Bax activation were essential for HBHA-induced apoptosis, as evidenced by a restoration of the viability of macrophages pretreated with N-acetylcysteine, a potent ROS scavenger, or transfected with Bax siRNA. HBHA is targeted to the mitochondrial compartment of HBHA-treated and *M. tuberculosis*-infected macrophages. Dissipation of the mitochondrial transmembrane potential (ΔΨ_m_) and depletion of cytochrome *c* also occurred in both macrophages and isolated mitochondria treated with HBHA. Disruption of HBHA gene led to the restoration of ΔΨ_m_ impairment in infected macrophages, resulting in reduced apoptosis. Taken together, our data suggest that HBHA may act as a strong pathogenic factor to cause apoptosis of professional phagocytes infected with *M. tuberculosis*.

## Author Summary

Cell death is a common outcome during infection with a number of pathogenic microorganisms. Therefore, defining the factors responsible for killing of host cells is important to uncovering mechanisms of pathogenesis. World-wide, two billon people are latently infected with *Mycobacterium tuberculosis*, which is still killing 2–3 million people each year. Heparin-binding hemagglutinin (HBHA) protein of *M. tuberculosis* is known to interact specifically with non-phagocytic cells and to be involved in dissemination from lungs to other tissues. Nevertheless, the role of HBHA in phagocytic cells such as macrophages, which are the first cells of the immune system to encounter inhaled pathogens, has been unknown. In the present study, we suggest HBHA as a critical bacterial protein for macrophage cell death. After *M. tuberculosis* infection or HBHA treatment of macrophages, HBHA targeted to mitochondria and then caused mitochondrial damage and oxidative stress, which eventually lead to apoptosis. A mutant of *M. tuberculosis* lacking HBHA induced less apoptosis with moderated mitochondrial damage. These experiments provide a candidate virulence factor which may be a novel target for tuberculosis treatment.

## Introduction

Tuberculosis remains a serious global problem, although many researchers have made a persistent effort for several decades. *Mycobacterium tuberculosis*, a major causative agent of pulmonary tuberculosis, is responsible for 1.8 million deaths per year worldwide [1]. Innate immune system plays a critical role in antimicrobial host response during the early stage of *M. tuberculosis* infection. Alveolar macrophages mediate innate immunity by phagocytosing pathogens and are the chief defense against *M. tuberculosis*, which can survive and replicate within phagocytes [2]. The course of tuberculosis rests on the outcome of the interaction between the bacterium and host macrophage. Therefore, a better understanding of these complex interactions is critical to controlling mycobacterial infection.

Many bacterial and viral pathogens utilize various strategies to manipulate host machinery to serve their own needs. Apoptotic cell death has been regarded as an innate cellular response to limit the multiplication of intracellular pathogens [3], although the precise mechanism of the direct antimicrobial action in infected macrophages undergoing apoptosis is unclear. Generally, infectious intracellular pathogens tend to prevent host cell apoptosis during an early stage of infection. However, they may also induce host cell apoptosis with a specific aim to subvert the host attack, such as immune and inflammatory response, at later stages [4], [5].

A number of reports have indicated that *M. tuberculosis* does indeed inhibit host cell apoptosis, while at the same time it induces pro-apoptotic signals. Recent studies showed that only virulent mycobacterial species can inhibit apoptosis induction in primary human alveolar macrophages [6], THP-1 [7], [8], and J774 macrophage cell lines [9]. Virulent *M. tuberculosis* reportedly induced the apoptotic death of host cells. For example, enhanced apoptotic response was detected in alveolar macrophages recovered from patients with pulmonary tuberculosis [10], [11]. Extensive apoptosis was also observed in caseating granulomas from lung tissue samples obtained from patients with tuberculosis [12], [13]. Several apoptosis-inducing factors of *M. tuberculosis*, such as 19-kDa glycolipoprotein (Rv3763) [14], PE_PGRS33 (Rv1818c) [15], ESAT6 (Rv3875) [16], and 38-kDa lipoprotein (Rv0934) [17] are reported.

Heparin-binding hemagglutinin adhesin (HBHA) is a 28-kDa multifunctional protein found on the surface and culture filtrates of mycobacteria. It has hemagglutination activity and binds to sulfated glycoconjugates such as heparin and dextran sulfate [18]. HBHA interacts specifically with non-phagocytic cells and is essential for the infection of lung epithelial cells and extrapulmonary dissemination of *M. tuberculosis* [18], [19]. Protective immunity induced by HBHA is observed in *M. tuberculosis*-infected mouse models, indicating that HBHA is a protective antigen [20]. Recent studies suggest that HBHA is a useful diagnostic marker for tuberculosis [21]. We also identified and characterized HBHA as a serologically active mycobacterial antigen in a previous study, whereby HBHA binds strongly to the immunoglobulin M of patients with tuberculosis [22]. Although HBHA function in mycobacterial pathogenesis has been extensively studied, the role of HBHA on professional phagocytes, such as macrophages, is still poorly understood.

The aim of the present study was to characterize the biological effects of *M. tuberculosis* HBHA on macrophages. We found that HBHA induced apoptosis in murine macrophages and investigated its underlying mechanism. Here, we show that HBHA treatment caused a loss of mitochondrial transmembrane potential (ΔΨ_m_) and the release of cytochrome *c* from purified mitochondria *in vitro*, as well as mitochondria of intact cells, and HBHA was efficiently targeted to mitochondria of macrophages.

## Results

### HBHA induces macrophage apoptosis via caspase activation

**Figure 1:**
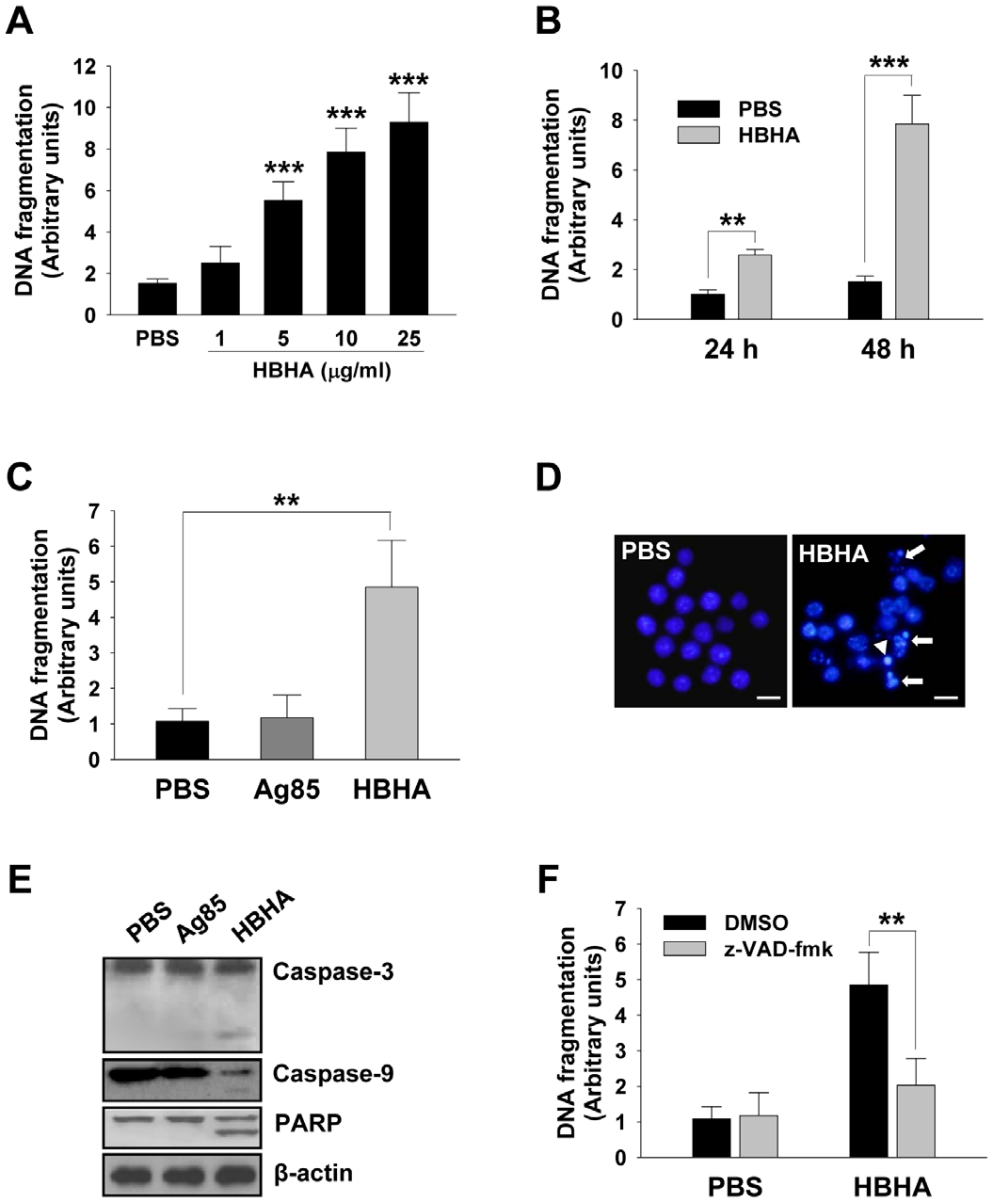
HBHA-induced macrophage apoptosis. (**A,B**) DNA fragmentation of RAW 264.7 cells incubated with the indicated concentrations (A) or 10 µg/mL (B) of HBHA for 24 or 48 h was measured by Cell Death Detection ELISA. (**C**) DNA fragmentation of BMDMs incubated with HBHA (10 µg/mL) or Ag85 (10 µg/mL) for 48 h was measured by Cell Death Detection ELISA. Values represent the mean OD_405_ ± SD of at least three experiments. ** *P*<0.01, *** *P*<0.001 RAW 264.7 cells treated with PBS *versus* those treated with HBHA. (**D**) RAW 264.7 cells were treated with HBHA (10 µg/mL) or PBS for 36 h, fixed, and stained with DAPI. Fragmented nuclei are indicated by arrows, and a condensed nucleus is denoted by an arrowhead. Scale bar, 10 µm. (**E**) BMDMs cell lysates exposed to HBHA for 36 h were immunoblotted against caspase-3, caspase-9, and PARP. To confirm equal protein loading, blots were re-probed with an antibody against β-actin. (**F**) BMDMs were incubated with PBS or HBHA (10 µg/mL) in the presence or absence of zVAD-fmk (50 µM). DNA fragmentation was measured as described above. Results are the mean ± SD of three independent experiments. ** *P*<0.01 between cells incubated with HBHA only and those incubated with HBHA+zVAD-fmk.

We first sought to determine whether HBHA could induce macrophage apoptosis. Apoptosis was assessed by quantifying DNA fragmentation, which is considered a hallmark of apoptosis, in the cytoplasmic fractions of dying cells using a commercially available ELISA kit. The incubation of RAW 264.7 cells with HBHA resulted in a significant increase in the release of oligonucleosomal fragments into the cytoplasm in both dose- and time-dependent manners as compared to compared to control cells (Figure 1A and 1B). Cell death was significantly greater in cells treated with HBHA as compared to buffer-treated control cells. As lactate dehydrogenase was not detected in the cell culture supernatant during HBHA treatment, the possibility that HBHA-induced death is necrosis was excluded (Figure S1). We used native antigen 85 complex (Ag85) as an unrelated control protein. The Ag85 of *M. tuberculosis* is the major secreted protein and fibronectin-binding protein, and shows strong immunoreactivity [23], [24]. Similar results were observed in bone marrow-derived macrophages (BMDMs); like PBS-treated BMDMs DNA fragmentation was not detected in Ag85-treated cells, whereas dramatic DNA fragmentation was observed in HBHA-treated cells (Figure 1C). HBHA-induced apoptosis was further confirmed by examining the nuclear morphology of dying cells using a fluorescent DNA-binding agent, 4′-6-diamidino-2-phenylindole (DAPI). As shown in Figure 1D, control cells treated with buffer had intact nuclei. In contrast, within 48 h of HBHA treatment, RAW 264.7 cells clearly exhibited condensed or fragmented nuclei indicative of apoptotic cell death. We further analyzed the caspase dependency of HBHA-induced apoptosis. Western blot analysis showed that the cleavage of caspase-3, caspase-9, and poly(ADP-ribose) polymerase (PARP) was evident in cells incubated with HBHA for 48 h (Figure 1E). Inhibition of caspases by a pan-caspase inhibitor, zVAD-fmk, attenuated the HBHA-induced DNA fragmentation, indicating that HBHA induces caspase-dependent apoptosis (Figure 1F). These results suggest that macrophages treated with HBHA undergo caspase-dependent apoptosis.

### HBHA causes a decrease in ΔΨ_m_

**Figure 2:**
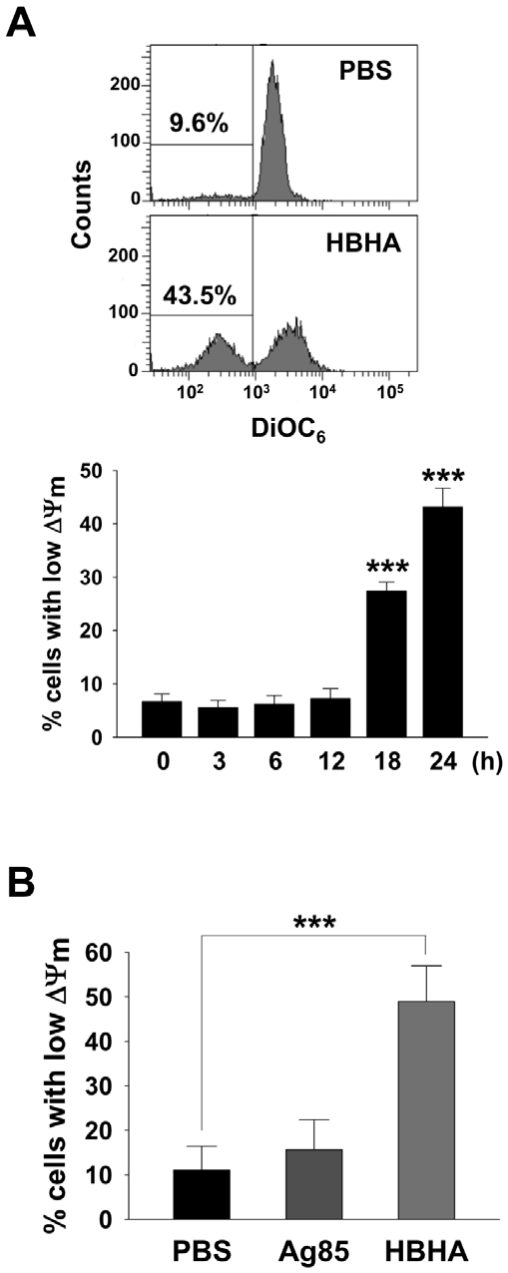
HBHA-induced the loss of ΔΨ_m_ in macrophages. RAW 264.7 cells (A) or BMDMs (B) were incubated with PBS, Ag85 (5 µg/mL), or HBHA (5 µg/mL) for the indicated time periods (A) or 24 h (B). Cells were washed and stained with DiOC_6_ (40 nM). The fluorescence activity of DiOC_6_ was determined by flow cytometry as described in the Materials and Methods. A shift in the cell population to the left indicates a loss of ΔΨ_m_. Histograms shown are representative of at least three independent experiments. Mean ± SD of three independent experiments is shown. *** *P*<0.001 cells treated with PBS *versus* those treated with HBHA.

The mitochondrion acts as a central executioner in response to apoptotic stimuli, allowing signals from various inputs to converge [25]. We investigated whether HBHA treatment affected the structural and biochemical integrity of mitochondria. Mitochondrial damage was assessed by examining mitochondrial ΔΨ_m_, which was determined by staining cells with 3,3′-Dihexyloxacarbocyanine (DiOC_6_), a dye that incorporates into mitochondria with intact membrane potential [26], for flow cytometric analysis. As shown in Figure 2A, a significant loss of ΔΨ_m_ was observed in RAW 264.7 cells incubated with HBHA as indicated by a decrease in DiOC_6_ intensity. Analysis of the time course for examination of ΔΨ_m_ onset showed a noticeable dissipation of ΔΨ_m_ after 18 h of HBHA treatment, which further decreased with time. A similar result was obtained in BMDMs incubated with HBHA (Figure 2B). These results suggest that mitochondrial damage appears as a subsequent event in the intracellular action of HBHA.

### HBHA induces Bax translocation to mitochondria and releases cytochrome c from mitochondria to the cytosol

**Figure 3:**
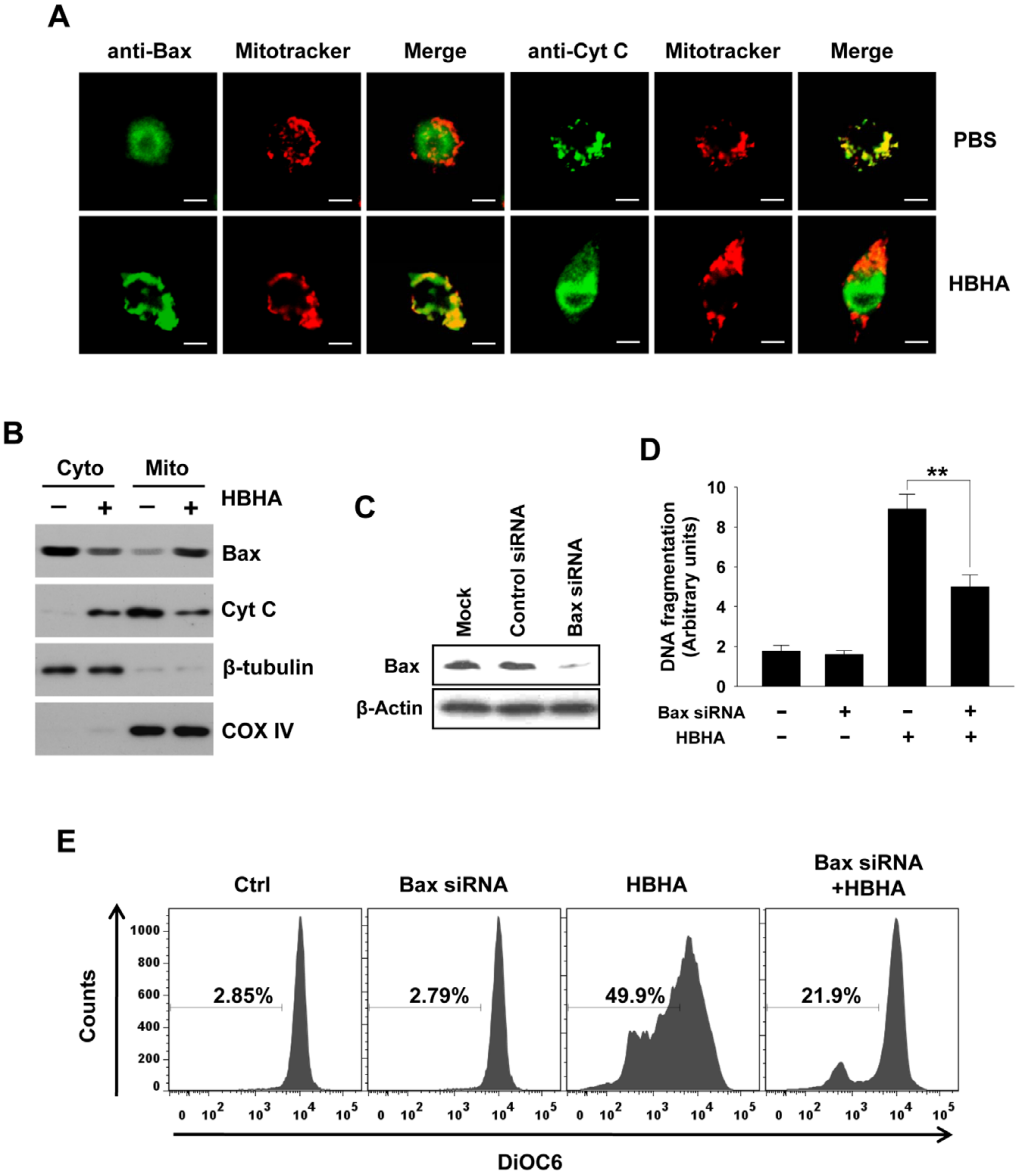
Bax translocation and cytochrome *c* release by HBHA treatment in macrophages. (**A**) RAW 264.7 cells were incubated with HBHA (10 µg/mL) or PBS for 20 h. Bax and Cytochrome *c* were stained with their antibodies and then an FITC-conjugated secondary antibody (green). Mitochondria were stained with Mitotracker Red (red). Cells were then imaged by confocal microscopy. Scale bar, 5 µm. (**B**) Mitochondrial and cytosolic fractions were prepared, and aliquots containing 20 µg of protein were subjected to Western blot analysis and probed with antibodies for Bax and cytochrome *c* (Cyt C) as described in the Materials and Methods. COX IV and β-tubulin were used as markers for the mitochondrial and cytosolic fractions, respectively. Results are representative of three independent experiments. (**C**) Western blot analysis of expression of Bax in RAW 264.7 cells transfected with liposomes only (Mock), nonspecific siRNA (Control siRNA), or Bax-specific siRNA. Western blotting was done using an antibody against Bax and an antibody against β-actin as a loading control. (**D,E**) Parental cells and Bax siRNA-transfected cells were treated with HBHA (10 µg/mL) for 24 h or 48 h. DNA fragmentation was determined by Cell Death Detection ELISA as described in Figure 1 (**D**). Loss of ΔΨ_m_ was assessed by DiOC_6_ retention assay as described in Figure 2 (**E**). Data are the mean ± SD from three separate experiments. ** *P*<0.01 between HBHA-treated parental cells and HBHA-treated cells transfected with Bax siRNA. Flow cytometric histograms are representative of three independent experiments.

Apoptosis at the mitochondrial level involves the oligomerization of the pro-apoptotic protein Bax [27], leading to permeabilization of the outer mitochondrial membrane (MOMP) and release of cytochrome *c* [28]. We performed immunocytochemistry to detect Bax translocation and cytochrome *c* release. An antibody recognizing the Bax N-terminus, which is exposed by the activation of Bax and its insertion into the mitochondrial membrane, was used. Figure 3A shows the translocation of Bax distributed evenly in the cytoplasm to the mitochondria in macrophages as evident by the colocalization of Bax with Mitotracker, a potential-sensitive dye specific for mitochondria. In PBS-treated cells, cytochrome *c* showed a punctate pattern that colocalizes with Mitotracker, whereas the faint signal for cytochrome *c* and the decreased colocalization with Mitotracker were detected in HBHA-treated cells, indicating cytochrome *c* release. These results were confirmed by performing subcellular fractionation and Western blot analysis (Figure 3B). HBHA caused a decrease in cytochrome *c* immunoreactivity in the mitochondrial fraction with a concomitant increase in the cytosolic fraction and vice versa for Bax immunoreactivity. Collectively, these findings suggest that the apoptotic effect of HBHA on macrophages is associated with cytochrome *c* release and Bax translocation. To determine whether Bax activation is necessary for HBHA-induced apoptosis, we knocked down the level of Bax by transfecting RAW 264.7 cells with Bax siRNA. The Bax protein level was significantly reduced in cells transfected with Bax siRNA; Bax protein in control siRNA-transfected cells was unchanged (Figure 3C). We then determined the effect of knockdown Bax on HBHA-induced apoptosis in RAW 264.7 cells. As shown in Figure 3D and 3E, HBHA-induced increase in DNA fragmentation was blocked and ΔΨ_m_ loss was restored by Bax knockdown, suggesting that Bax activation is required for HBHA-induced macrophage apoptosis.

### Reactive oxygen species (ROS) are required for apoptosis induced by HBHA

**Figure 4:**
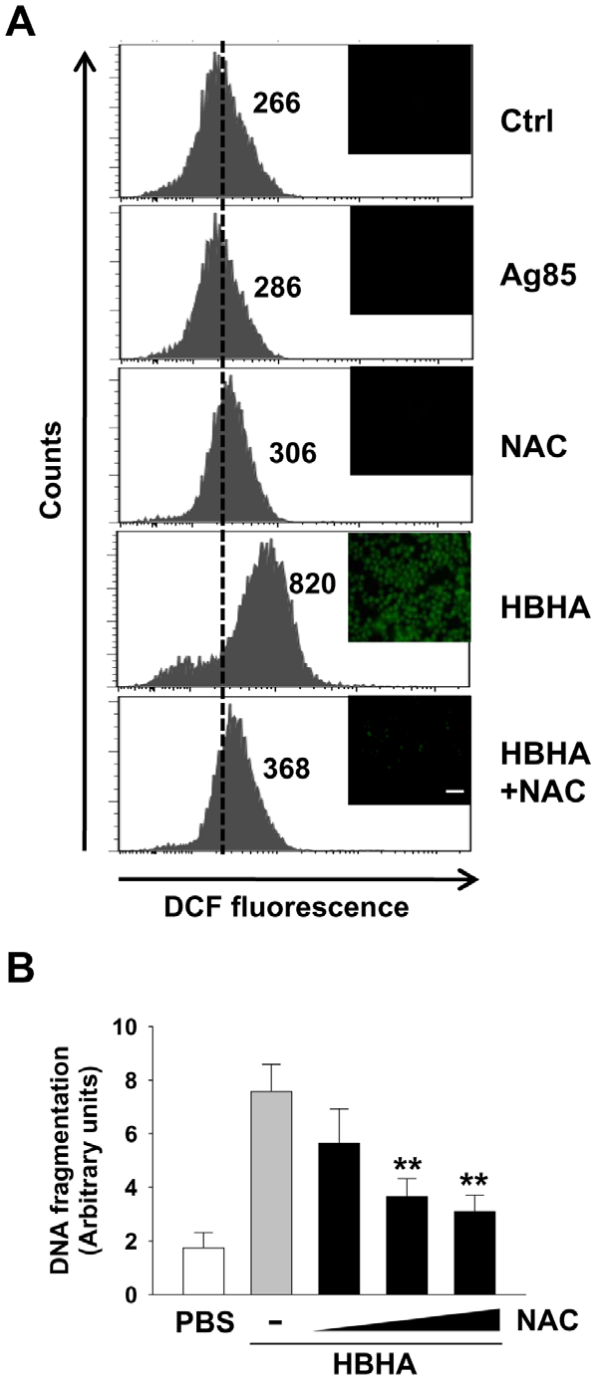
HBHA-induced ROS production and ROS scavenger effect on DNA fragmentation in macrophages. (**A**) RAW 264.7 cells were treated with PBS, Ag85 (5 µg/mL), or HBHA (5 µg/mL) in the presence or absence of NAC (10 mM) for 24 h. ROS levels were measured by flow cytometry and fluorescent microscopy after DCF treatment. The number indicated for each histogram represents the mean DCF fluorescence from one of three independent experiments that gave similar results. Scale bar, 100 µm. (**B**) RAW 264.7 cells were incubated with HBHA (10 µg/mL) alone or pretreated with NAC in increasing concentration (0 to 20 mM). DNA fragmentation was measured by Cell Death Detection ELISA as described in Figure 1. All values are the mean ± SD of three separate sets of experiments. ** *P*<0.01 compared to the control treated with HBHA alone.

Enhanced reactive oxygen species (ROS) production, characteristic of early apoptotic events, can be both a cause and a consequence of changes in ΔΨ_m_ [26], [29]. To examine the involvement of ROS generation on HBHA effects in macrophages, the oxidation of DCF was monitored by flow cytometry and fluorescent microscopy (Figure 4A). Compared to PBS or Ag85, HBHA significantly induced the increase of intracellular hydroperoxide in macrophages. To determine the requirement of ROS increase in HBHA-induced apoptosis, the effect of HBHA alone or in combination with N-acetylcysteine (NAC), a general ROS scavenger, on DNA fragmentation was assessed. NAC pretreatment effectively inhibited HBHA-induced DNA fragmentation (Figure 4B) as well as ROS production, suggesting that ROS increase is essential for the apoptotic response caused by HBHA.

### HBHA is targeted to the mitochondria

**Figure 5:**
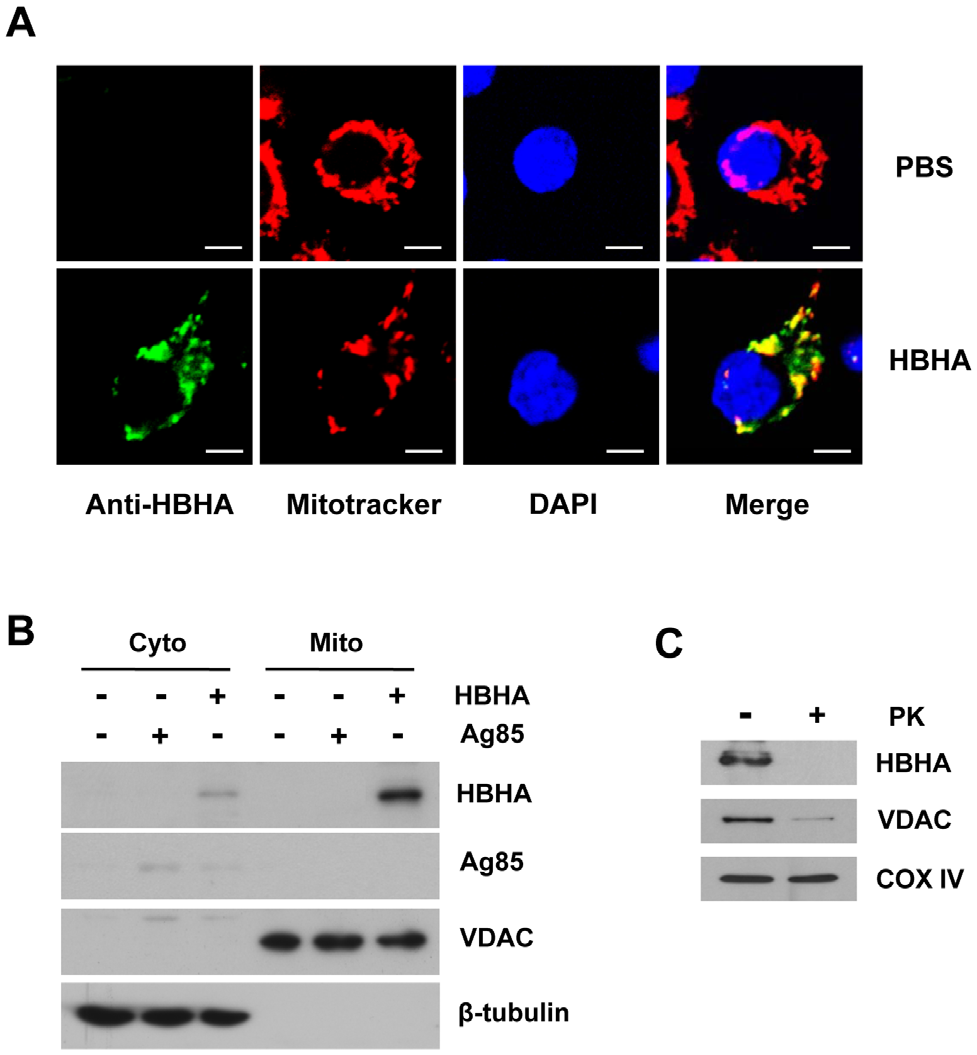
Subcellular localization of HBHA in macrophages. RAW 264.7 cells were exposed to HBHA (5 µg/mL), Ag85 (5 µg/mL), or an equal volume of PBS for 12 h. (**A**) Representative confocal images of HBHA (green) and Mitotracker (red) in PBS- or HBHA-treated cells. DNA was visualized with DAPI (blue). Yellow in merged images indicates co-localization. Scale bars, 5 µm. (**B**) Mitochondrial and cytosolic fractions were separated from cell lysates and examined by Western blot using specific antibodies against HBHA, VDAC, and β-tubulin. (**C**) Isolated mitochondria from cells exposed to HBHA were incubated in the presence or absence of proteinase K (200 µg/mL) for 30 min on ice. Samples were subjected to Western blot for HBHA and VDAC as described above. COX IV was used as a loading control.

Studies have suggested that some infectious intracellular pathogens regulate apoptosis of their host cells by targeting proteins to mitochondrial membranes that either induce or inhibit MMP [30]. We addressed the question of where HBHA is localized in mitochondria of HBHA-treated cells. Therefore, the possibility that HBHA interacts with the mitochondrial compartment was examined. Confocal microscopic analysis revealed the presence of HBHA in the mitochondria of HBHA-treated cells, as evidenced by a significant overlap between HBHA and Mitotracker (Figure 5A). Subcellular fractionation and Western blot analysis consistently showed that large amounts of HBHA were detected in the mitochondrial fraction, but not in the cytosolic fraction, where little HBHA was observed (Figure 5B). In contrast, the minimum of Ag85 were detected in cytoplasmic fraction of macrophage treated with Ag85, suggesting that it is not able to pass through plasma membrane. Furthermore, to determine whether HBHA was imported into mitochondria, we isolated mitochondria from cells treated with HBHA. The purified mitochondria were subsequently digested with proteinase K. As shown in Figure 5C, HBHA disappeared in mitochondria digested with proteinase K, indicating that HBHA adheres to the outer membrane of the mitochondria.

### HBHA induces the release of cytochrome c and the loss of ΔΨ_m_ in isolated mitochondria

**Figure 6:**
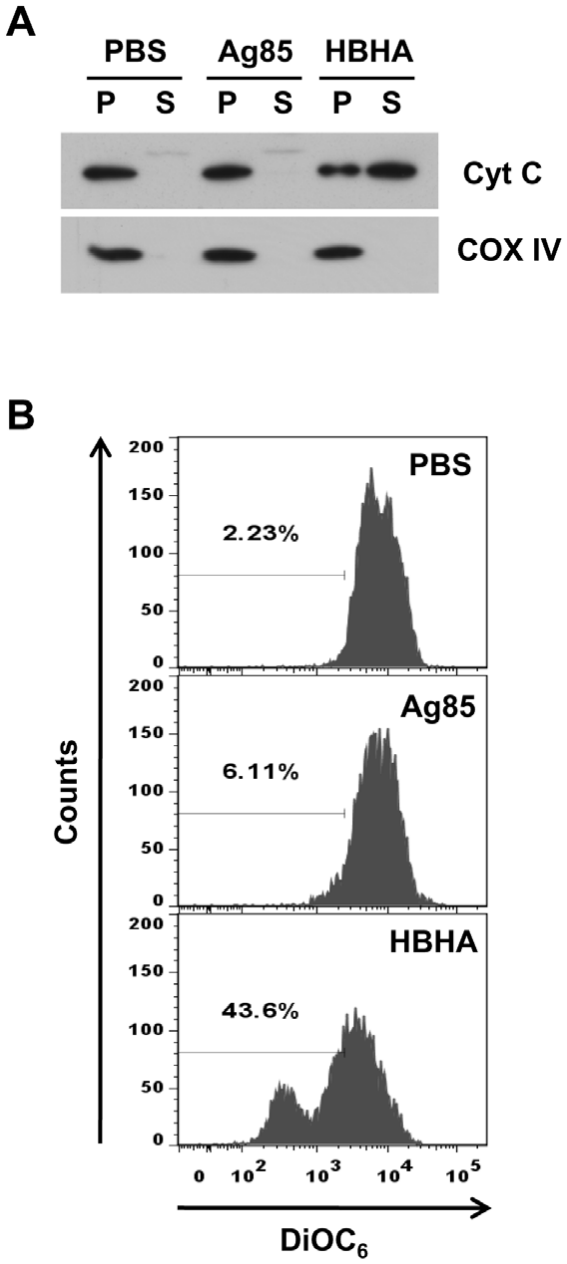
Effect of HBHA on cytochrome *c* release and ΔΨ_m_ dissipation in mitochondria *in vitro*. (**A**) Mitochondria were purified from RAW 264.7 cells and treated with HBHA (5 µg/mL), Ag85 (5 µg/mL), or an equal volume of PBS for 1 h at 37°C. The pellet (P) and the supernatant (S) of samples were then separated by SDS-PAGE and analyzed by Western blotting using antibodies against cytochrome *c* and COX IV. Results are from one of two independent experiments. (**B**) Purified mitochondria were stained with DiOC_6_ for 20 min at 37°C and analyzed by flow cytometry. Data are representative histogram plots taken from three separate experiments.

We next determined whether HBHA induced cytochrome *c* release from isolated mitochondria. As shown in Figure 6A, isolated mitochondria from RAW 264.7 cells released cytochrome *c* after HBHA treatment, whereas the buffer control or Ag85 did not stimulate this release in a cell-free assay. We also examined the effect of HBHA on the collapse of membrane potential in purified mitochondria. For this, mitochondria incubated with HBHA were stained with DiOC_6_, and the fluorescence intensity was monitored by flow cytometry (Figure 6B). A significant shift to a lower intensity was observed in mitochondria treated with HBHA as compared to buffer control or Ag85, indicating the decrease in ΔΨ_m_. These data provide evidence that similar to the event that occurs in macrophages, HBHA can solely induce mitochondrial damage in a cell-free system, indicating that Bax translocation to mitochondria is not essential for of ΔΨ_m_ loss and cytochrome *c* release.

### HBHA partially localizes to mitochondria during *M. tuberculosis* infection and impacts viability of infected macrophages

**Figure 7:**
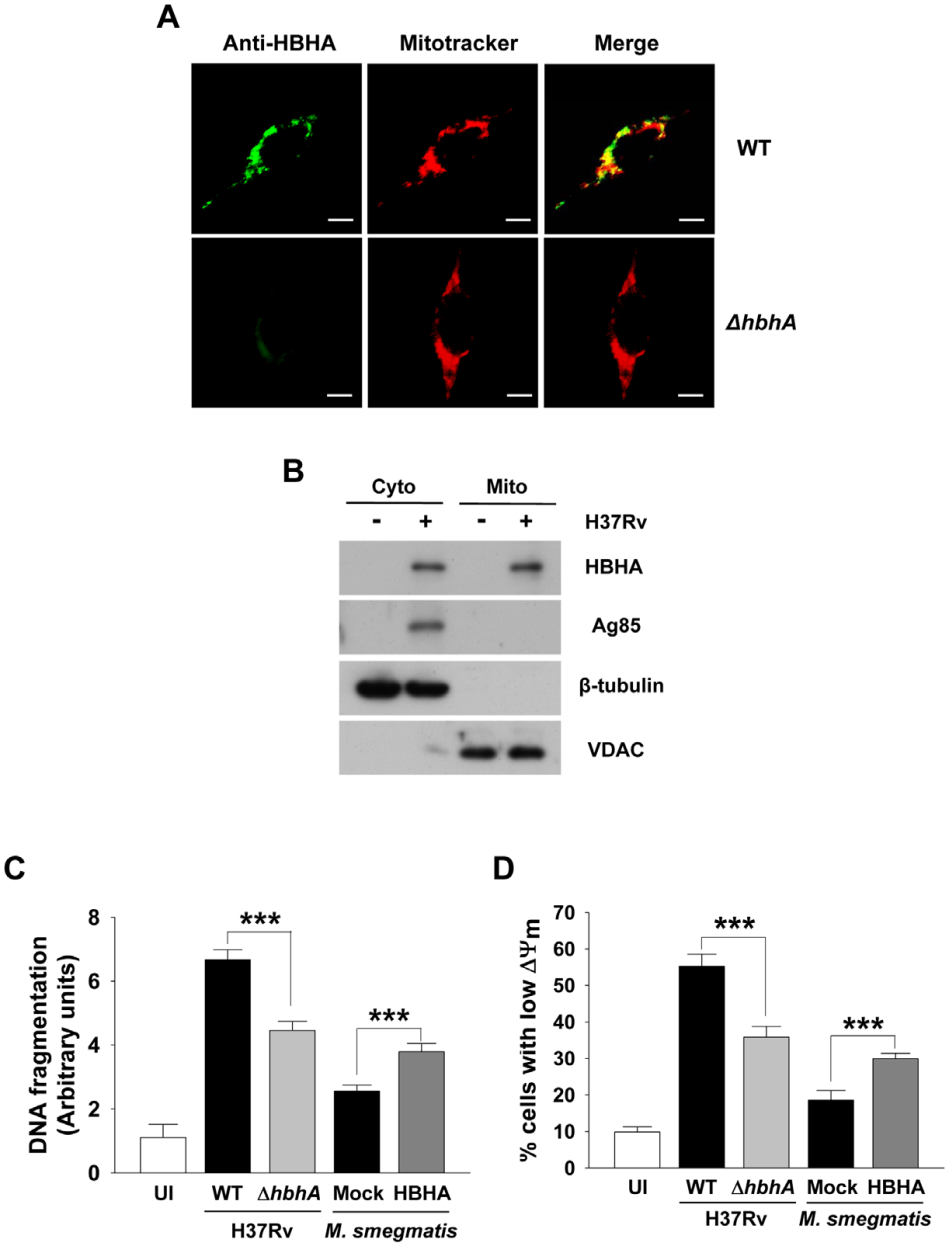
Disruption of *hbhA* decreases apoptosis and ΔΨ_m_ dissipation in macrophages infected with *M. tuberculosis*. (**A**) RAW 264.7 cells were infected with *M. tuberculosis* 103 wild-type (WT) and mutant (*ΔhbhA*) at a multiplicity of infection (MOI) of 1 for 12 h. Cells were double stained with Mitotracker and HBHA-specific antiserum followed by an Alexa 488-conjugated secondary antibody. Scale bar, 5 µm. (**B**) BMDMs were infected with *M. tuberculosis* at an MOI of 1 for 12 h. Mitochondrial and cytosolic fractions were separated from cell lysates and examined by Western blot using specific antibodies against HBHA, Ag85, VDAC, and β-tubulin. (**C,D**) BMDMs were infected with *M. tuberculosis* wild-type (WT) and mutant (*ΔhbhA*) or *M. smegmatis* ectopically expressing HBHA (HBHA) or empty plasmid (Mock) at an MOI of 5 for 24 h. DNA fragmentation (C) and ΔΨ_m_ (D) were measured as described in Figure 1 and 2, respectively. Data are the mean ± SD from six separate experiments. *** *P*<0.001 cells infected with *M. tuberculosis* WT *versus* with mutant strain and cells infected with *M. smegmatis* expressing HBHA *versus* with Mock transformant.

HBHA is a secreted protein in *M. tuberculosis* as well as a surface-associated protein [18]. To examine whether HBHA is also transported to mitochondria during *M. tuberculosis* infection, BMDMs were infected with H37Rv wild type and mutant disrupted in *hbhA*. Immunofluorescence microscopy of infected cells revealed that a part of HBHA colocalized with mitochondria (Figure 7A). Purified mitochondrial fraction of these cells contained a considerable amount of HBHA protein, although a large portion of HBHA were observed in cytosolic fraction (Figure 7B). These findings demonstrate that HBHA is efficiently transported to mitochondria of infected macrophages. To analyze the effects of HBHA on macrophages in the context of the bacterium as a whole, we compared the relative ability of *M. tuberculosis* H37Rv wild type and mutant disrupted in *hbhA* to induce apoptosis and ΔΨ_m_ collapse in macrophages. A reduced DNA fragmentation and an increased intact mitochondria were observed in BMDMs infected with mutant strain compared to its parent (Figure 7C and 7D), which was noticeable when macrophages were infected at MOIs of 5 and 10 but not at an MOI of 25 (Figure S2A). On the other hand, there was no significant difference in LDH release between cells infected with two strains (Figure S2B). Similarly, more significant DNA fragmentation and ΔΨ_m_ loss were detected in cells infected with *M. smegmatis* ectopically expressing HBHA compared to cells infected with the *M. smegmatis* control.

### HBHA has no influence on the viability of A549 cells

**Figure 8:**
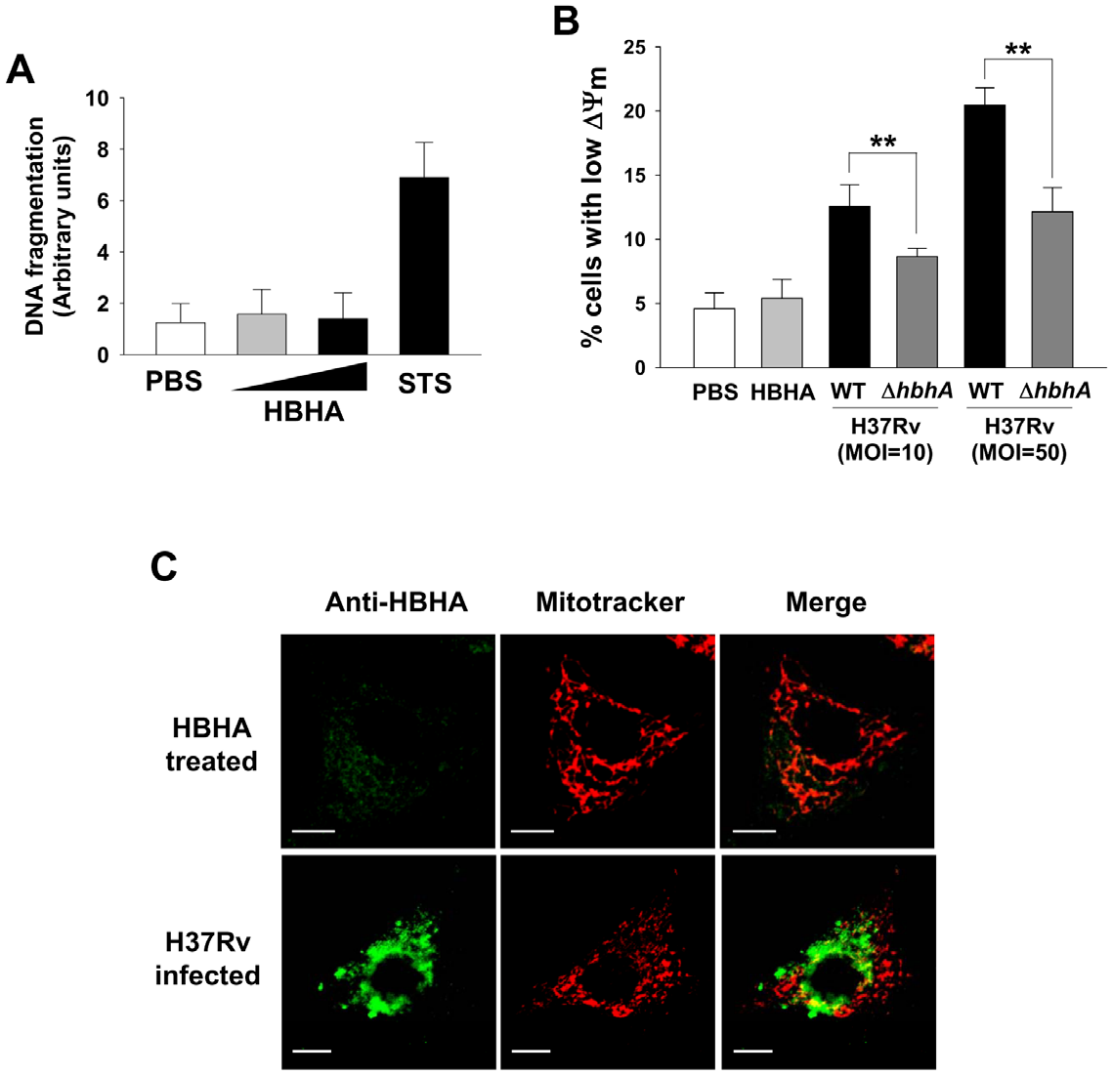
HBHA does not affect the viability of A549 cells. (**A**) DNA fragmentation of A549 cells incubated with HBHA (5 or 25 µg/mL) or PBS for 48 h was measured by Cell Death Detection ELISA. Staurosporine (STS, 1 µM, 4 h) was used as a positive control for apoptosis. Results are the mean ± SD of three independent experiments. (**B**) A549 cells were incubated with 25 µg/mL of HBHA or infected with *M. tuberculosis* wild-type (WT) and mutant (*ΔhbhA*) at an MOI of 10 or 50 for 24 h. Cells were stained with DiOC_6_. As described in Figure 2, the loss of ΔΨ_m_ was evaluated by flow cytometry. All values are the mean ± SD of three separate sets of experiments. ** *P*<0.01 between cells infected with WT and mutant strains. (**C**) Representative confocal images of HBHA (green) and Mitotracker (red) in A549 cells treated with HBHA (10 µg/mL) (upper) or *M. tuberculosis* at an MOI of 10 (lower). Scale bar, 10 µm.

HBHA is involved in the interaction of mycobacteria with alveolar epithelial cells [19]. To determine whether these cells exposed to HBHA undergo apoptosis, human type II A549 pneumocytes were treated with purified HBHA for 48 h. As shown in Figure 8A and 8B, neither DNA fragmentation nor ΔΨ_m_ collapse was observed in HBHA-treated A549 cells. Immunofluorescent microscopy showed that a very faint green signal was detected in A549 cells incubated with HBHA, indicating that HBHA enters A549 cells much less efficiently (Figure 8C, upper panels). To confirm this issue, A549 cells were infected with *M. tuberculosis* wild type and *hbhA* deficient strains. Like experiments conducted in macrophages, *M. tuberculosis* infection led to severe ΔΨ_m_ dissipation, accompanied by the partial presence of HBHA in mitochondrial compartments (Figure 8B and 8C, lower panels). In contrast, a decrease in the percentage of cells displaying loss of ΔΨ_m_ was observed in A549 cells infected with the *hbhA* deficient strain (Figure 8B). These data suggest that cell entry and targeting to mitochondria of HBHA are essential for ΔΨ_m_ loss and apoptotic response.

## Discussion

Programmed cell death is emerging as a major effect of bacterial pathogenesis. Numerous studies have shown that *M. tuberculosis* infection can increase the rate of macrophage apoptosis [31], [32]. Pro-apoptotic activities of a growing number of mycobacterial components have recently been described [14]–[17]. Nevertheless, data regarding the identities of the mycobacterial molecules involved and the underlying apoptotic mechanism are still scarce. We showed here that intracellular HBHA is targeted to mitochondria in murine macrophages, which leads to ΔΨ_m_ dissipation and eventual apoptosis. Although the possibility that HBHA may interact with cytosolic molecules or other cell compartments cannot be ruled out completely, these connections clearly appear to be insignificant. To our knowledge, the present study is the first description of a mycobacteria-encoded protein stimulating apoptotic cell death via a mitochondria-dependent pathway in macrophages.

*M. tuberculosis* HBHA is a protein that is both surface-associated and secreted. HBHA is involved in the binding of *M. tuberculosis* to type II pneumocytes, but not to professional phagocytes such as macrophages, and is required for the dissemination of tubercle bacilli from the lungs to other tissues [19]. In this respect, its impact on macrophages has received relatively little attention. However, HBHA was recently demonstrated to have the capacity to bind to complement component C3, and recombinant HBHA was found to mediate the attachment of latex beads to murine macrophage-like cells in both C3-dependent and -independent manners [33]. *M. tuberculosis* can bind to the complement receptors and is subsequently introduced into the phagocytic cell [34]. These results raise the possibility of the interaction between HBHA and macrophages during mycobacterial infection.

Mitochondria are central organelles in which a variety of key events in apoptosis occur, including the release of cytochrome *c*, changes in electron transport, ΔΨ_m_ collapse, altered cellular oxidation–reduction, and participation of pro- and anti-apoptotic Bcl-2 family proteins [26]. Presently, mitochondria are regarded as the targets for the manipulation of many bacterial and viral pathogens determining the fate of infected host cells [35]. Moreover, mitochondrial damage has been suggested to play a critical role in the outcome of macrophage infection with *M. tuberculosis* [36]. These findings offer the potential of mycobacterial components for the regulation of programmed cell death at the mitochondrial level.

MMP is regulated by endogenous molecules, including Bcl-2 family members such as Bax [37]. The Bax present in the cytosol under normal conditions fosters the loss of ΔΨ_m_ and releases cytochrome *c* and apoptosis-inducing factor (AIF) from mitochondria after its introduction into the mitochondrial compartment [26]. Indeed, mitochondrial translocation of Bax was observed in macrophages treated with HBHA, and the interaction of HBHA with mitochondria resulted in cytochrome *c* release in murine macrophages. However, Bax translocation may not be essential for mitochondrial dysfunction by HBHA, as evidenced by a mitochondrial cell-free assay in which HBHA caused ΔΨ_m_ loss and cytochrome *c* release *in vitro*. Not surprisingly, we observed the activation of caspases 3 and 9 and subsequent cleavage of PARP after incubation of macrophages with HBHA. In contrast, we found no evidence of cytosolic or nuclear translocation of AIF induced by HBHA (data not shown), indicating that it is not involved in HBHA-induced cell death. ROS generation with ΔΨ_m_ modulation and caspase-9 activation is known to be a major component of the mitochondrial pathway of apoptosis [38]. ROS are predominantly produced in the mitochondria and lead to the modulation of ΔΨ_m_, which finally results in apoptosis [39]. Our results indicate that HBHA induces macrophage apoptosis through ROS generation and ΔΨ_m_ collapse, suggesting that these play an essential role in HBHA-induced apoptosis.

Our results indicate that cellular entry is essential for mitochondria-mediated apoptotic effect of HBHA, although the mechanism by which HBHA internalized by host cells remains unresolved. In A549 cells infected with *M. tuberculosis* but not cells incubated with purified HBHA, the severe ΔΨ_m_ collapse and the presence of intracellular HBHA in mitochondrial compartment were observed. There was a significant increase in the percentage of cells with intact ΔΨ_m_, when A549 cells were infected with the mutant strain lacking HBHA gene. We cannot rule out that these results might come from decreased number of mycobacteria in cells, because invasion of A549 cells, but not macrophages, by HBHA-deficient strain compared with parental strain was reduced [19]. Moreover, HBHA induced ΔΨ_m_ loss and cytochrome *c* release in purified mitochondria from not only RAW 264.7 cells but also mouse liver (data not shown). Thus, no impact on viability of epithelial cells treated with HBHA might be due to the absence of intracellular this protein.

What host molecules physically and functionally interact with intracellular HBHA and how do they then induce mitochondrial dysfunction? In the present study, proteinase K digestion *in vitro* showed that intracellularly inserted HBHA is attached to the mitochondrial surface but is not imported into mitochondria, indicating that HBHA probably interacts with integral outer membrane molecules. Several mitochondria-targeted proteins encoded by pathogens interact with voltage-dependent anion channel (VDAC). The porin B from *N. meningitidis* is a VDAC-targeted protein [40]. Hepatitis B virus X protein also co-localizes to mitochondria where it interacts with a particular VDAC isoform, HVDAC3 [41]. Anti-apoptotic members of the Bcl-2 family, such as Bcl-2 and Bcl-x_L_, are located in mitochondrial membranes where they inhibit cytochrome *c* release from mitochondria and thereby prevent downstream caspase activation. Pro-apoptotic members of the Bcl-2 family, such as Bax, can translocate into mitochondria and induce MMP [42]. These Bcl-2-like proteins can be prominent targets of bacterial proteins [30], [43]. Recombinant HBHA used in the present study was a His-tagged fusion protein. To determine the interaction between HBHA and VDAC or the Bcl-2 family proteins, HBHA and interacting molecules were purified by Ni-NTA affinity chromatography, followed by immunoblotting against them. However, HBHA showed no direct interaction with VDAC or Bcl-2 family members (data not shown). In addition, the possibility of HBHA nonspecific binding to mitochondria cannot be excluded. The C-terminal region of HBHA contains several cationic lysine-rich repeats where methylation can occur [44]. This region may work like natural antibiotic peptides which form cationic residues on one end and interact with anionic molecules such as phospholipids to disrupt negatively charged membranes and result in apoptosis [45].

Virulent *M. tuberculosis* induces necrosis of the infected macrophages by inhibiting the repair process of plasma membrane; this leads to cellular lysis and reinforces the spreading to the adjacent infection sites [46]–[48]. Recent reports suggested that high intracellular burden of virulent *M. tuberculosis* induces host cell death via a new caspase-independent apoptotic pathway involved in the bacterial escape and extracellular replication [49], [50]. Because gain of function mutation in HBHA enhanced the apoptogenic potency of *M. smegmatis* (Figure 7C), it is plausible that HBHA may be the factor that allows *M. tuberculosis* to escape from the infected macrophages at high intracellular burden. However, similar levels of apoptosis were observed between macrophages infected with *M. tuberculosis* H37Rv wild type and mutant disrupted in *hbhA* at an MOI of 25 but not low MOIs (Figure S2A). Further, HBHA deficiency had no influence on the macrophage necrosis caused by *M. tuberculosis* at both low and high MOIs (Figure S2B), indicating no involvement of HBHA in bacterial escape from the macrophages at an early stage of infection.

Studies on the comparison of virulent and attenuated mycobacterial strains have demonstrated that the latter has much stronger apoptotic activity in macrophages. This concept is supported by the identification of genes that inhibit apoptosis of host cells [51]–[53]. In this sense, our claims that HBHA targets to the mitochondria of host cells in the induction of apoptosis may be confusing. However, there is cumulative evidence suggesting that virulent *M. tuberculosis* induces host cell apoptosis. Furthermore, the transcriptional profiling of cells infected with virulent *M. tuberculosis* showed increases in the expression of both pro- and anti-apoptotic genes [54], [55]. Collectively, it is highly likely that *M. tuberculosis* infection results in pro- and anti-apoptotic response of host cells. The final outcome may depend on the nature and activation status of the host cell. Although the pro-apoptotic response is inarguably beneficial to the host, it may provide a favorable circumstance for the induction of necrotic cell death and subsequent bacterial escape to the adjacent cells, which may provide a clue for HBHA function during *M. tuberculosis* infection [46], [49], [50].

Taken together, the present study suggests the possibility that the *M. tuberculosis* HBHA may be an apoptosis-inducing factor of mycobacteria, although the molecular mechanism by which HBHA causes loss of ΔΨ_m_ remains unknown. Future work should focus on the exploration of host targets of HBHA and the mechanism by which HBHA modulates ΔΨ_m_ and cytochrome *c* release in detail, as well as identification of the HBHA domain essential for its activity in mitochondrial dysfunction.

## Materials and Methods

### Ethics statement

All animal procedures were approved by the Institutional Animal Care and Use Committees of Chungnam National University (Permit Number: 2010-3-9). All animal experiments were performed in accordance with Korean Food and Drug Administration (KFDA) guidelines.

### Reagents and antibodies

Antibodies against caspase-3, caspase-9, and VDAC were purchased from Cell Signaling Technology Inc (Beverly, MA). The anti-PARP and anti-β-actin, anti-Bax, and anti-Tom40 antibodies were obtained from Santa Cruz Biotechnology (Santa Cruz, CA). Antibodies against cytochrome *c* (for immunofluorescence, clone 6H2.B4; for Western blot analysis, clone 7H8.2C12) were acquired from BD Pharmingen (San Diego, CA), and the anti-cytochrome oxidase subunit IV (COX IV) antibody was purchased from Abcam (Cambridge, UK). Dichlorodihydrofluorescein diacetate (H_2_DCFDA), DAPI, and DiOC_6_ were obtained from Molecular Probes (Eugene, OR) and zVAD-fmk and NAC were purchased from Calbiochem (San Diego, CA).

### Recombinant HBHA protein, Native Ag85 protein, anti-HBHA, and mycobacterial strains

*Mycobacterium smegmatis* strains, recombinant HBHA protein from *M. smegmatis*, and antiserum to HBHA were produced and prepared as described previously [22]. Ag85 was purified from the culture filtrate protein of *M. tuberculosis* H37Rv (ATCC 27294), as previously described by Lim et al [24]. Parental and mutant (*hbhA* deletion) *Mycobacterium tuberculosis* 103 were kindly provided by Dr. Camille Locht (Institut Pasteur de Lille, Lille, France) [19]. HBHA proteins were used in experiments after lipopolysaccharide (LPS) inactivation with polymyxin B (Invivogen, San Diego, CA), a known pharmacological antagonist of LPS.

### Cell culture

RAW 264.7 murine macrophage cell line and A549 human alveolar epithelial cell line were cultured in Dulbecco's modified Eagle's medium (DMEM; Lonza, Walkersville, MD) supplemented with 10% fetal bovine serum (FBS; Hyclone, Logan, UT), 1% HEPES, and 1% l-glutamine at 37°C with 5% CO_2_. BMDMs were obtained from 6–8-week-old female C57BL/6 mice. Briefly, bone marrow cells from the femur and tibia were cultured in DMEM that contained 2 mM l-glutamine, 100 U/mL penicillin, 100 µg/mL streptomycin, 10% FBS, and 25 ng/mL recombinant mouse M-CSF (R&D system, Minneapolis, MN) at 37°C with 5% CO_2_. After 4 days, non-adherent cells were removed and differentiated macrophages were incubated in antibiotic-free DMEM until use.

### Bax siRNA transfection

One day before transfection, RAW 264.7 cells were plated and grown at 37°C to 70% confluency in complete medium without antibiotics in 6 well plates. One micrograms of a Bax siRNA (Bioneer, Deajeon, Korea, sense: CCGGCGAAUUGGAGAUGAA; anti-sense: UUCAUCUCCAAUUGGCCGG) or a noncomplementary siRNA were transiently transfected into RAW 264.7 using Lipofectamine 2000 transfection reagent (Invitrogen, Carlsbad, CA, USA*)*, according to the manufacturer's instructions.

### DNA fragmentation assay (Apoptosis ELISA)

Cells were seeded in 96-well flat-bottom culture plates. After incubation with recombinant HBHA proteins, cells were collected, washed with PBS, and processed for quantification of cytoplasmic histone-associated DNA fragments formed during apoptosis using an enzyme-linked immunosorbent assay (Cell Death Detection ELISA PLUS; Roche Diagnostic) according to the manufacturer's instructions.

### Lactate dehydrogenase (LDH) assay

The release of LDH from RAW 264.7 cells incubated with recombinant HBHA or from BMDMs infected with *M. tuberculosis* was measured using a Cytotoxicity Detection Kit plus (Roche, Indianapolis, IN) according to the manufacturer's protocol. Relative cytotoxicity was calculated using the following equation: Cytotoxicity (%)  =  % of LDH released from the infected cells/maximum LDH released.

### Assessment of ΔΨ_m_

ΔΨ_m_ was assessed by measuring retention of the lipophilic cationic dye DiOC_6_ in mitochondria. Cells were harvested and incubated in a DiOC_6_ solution (10 nM in fresh medium) for 20 min at 37°C in the dark. The cells were then washed and resuspended in PBS. Immediately after PBS washing, ΔΨ_m_ was measured by sorting the cells using FACSCanto (BD Biosciences). Dead cells were excluded by forward and side-scatter gating. Data were acquired by analyzing an average population of 10 000 cells using CELLQuest software (BD Biosciences).

### Immunofluorescence microscopy

Cells were seeded onto glass coverslips in 12-well plates. Nuclear changes were analyzed by DAPI staining. After cells were incubated with HBHA for the indicated times, they were fixed with 4% paraformaldehyde and incubated with DAPI (10 µg/mL) for 10 min in the dark. The nuclei of stained cells were visualized using an Olympus BX50 fluorescence microscope (Olympus Optical Co., Hamburg, Germany). To determine the localization of cytochrome *c* or Bax, cells treated with HBHA were incubated in pre-warmed medium containing 100 nM of Mitotracker Red (Molecular Probes), fixed in 4% paraformaldehyde, permeabilized with 0.1% Triton X-100, and then stained with anti-cytochrome *c* or anti-Bax and Alexa-488-conjugated secondary antibody (Jackson Immuno Research Laboratories) before confocal microscopy. The subcellular localization of HBHA was analyzed using a confocal microscope (LSM510 META; Carl Zeiss). The cells incubated with Mitotracker Red were fixed, permeabilized, and stained with an anti-HBHA antibody followed by a fluorophore-conjugated antibody (anti-mouse IgG Alexa-488). After DAPI staining, cells were imaged with a confocal microscope.

### Mitochondrial and cytosolic fractionation

Subcellular fractionation was performed as previously described [56]. Briefly, cells were incubated on ice for 5 min in 100 µL of ice cold CLAMI buffer (200 mM sucrose, 70 mM KCl, 200 µg/mL digitonin in PBS) and centrifuged at 1,000 × *g* for 5 min at 4°C. The supernatants (cytosolic fractions) were stored at −80°C and the pellets were resuspended in 50 µL of IP buffer (50 mM Tris-Cl, pH 7.4, 150 mM NaCl, 2 mM EDTA, 2 mM EGTA, 0.2% Triton X-100, 0.3% NP-40) containing protease inhibitor cocktail (Roche Diagnostics Corporation, Indianapolis, IN) and incubated on ice for 10 min. The samples were centrifuged at 10 000 × *g* for 5 min at 4°C and the supernatants (mitochondrial fractions) were stored at −80°C until use in further experiments.

### Immunoblot analysis

Cells were detached, centrifuged, and lysed in lysis buffer (10 mM Tris, pH 7.4, 5 mM EDTA, 150 mM NaCl, 1% Triton X-100, 1 mM PMSF, protease inhibitor cocktail). Protein concentrations were determined with the Bradford assay and 30 µg of protein was separated with SDS-PAGE, followed by electrotransfer to a nitrocellulose membrane (Hybond-ECL; Amersham Pharmacia Biotech). The blots were probed with primary antibodies at optimized concentrations followed by horseradish peroxidase-conjugated secondary antibodies. The enhanced chemiluminescence system (ECL; Amersham/GE Healthcare) followed by exposure to chemiluminescence film was used to visualize proteins.

### Measurement of ROS

Intracellular ROS were evaluated through staining cells with H_2_DCFDA. Cells were incubated in 10 µM H_2_DCFDA for 30 min at 37°C, washed, and detached. Resuspended cells were washed and immediately analyzed by flow cytometry using FACSCanto. At least 10,000 cells per sample were analyzed using CellQuest Pro acquisition and analysis software.

### Mitochondrial cell-free assay

Mitochondria were isolated from 1 × 10^8^ RAW 264.7 cells as described previously [57]. Briefly, cells were harvested by centrifugation at 600 × *g* and resuspended in ice-cold IB buffer (10 mM Tris-MOPS, 200 mM sucrose, 1 mM EGTA/Tris, pH 7.4). All subsequent centrifugations were performed at 4°C. The cells were then homogenized with 35 strokes in a glass potter after incubation for 10 min on ice. Cell debris was removed by centrifugation at 600 × *g* for 10 min, and then the supernatant was centrifuged for 10 min at 7 000 × *g* to precipitate mitochondria. The pellet was then resuspended in EB buffer (10 mM Tris-MOPS, 125 mM KCl, 100 µM EGTA/Tris, 1 mM KH_2_PO_4_, pH 7.4). An aliquot of the preparation was incubated with HBHA for 1 h at 37°C and centrifuged for 10 min at 7 000 × *g*. The pellet containing mitochondria was resuspended in the same buffer and stained with DiOC_6_. An average population of 50 000 mitochondria was analyzed by flow cytometry. Alternatively, the proteins contained in the supernatant were concentrated with by ultrafiltration using a 3-kDa cutoff Centricon device (Amicon, Millipore, Bellerica, MA). Immunoblot analysis for cytochrome *c* was performed as described above.

### Statistical analysis

The data represent the mean ± standard deviation (SD) from at least three independent experiments. Statistical analyses were performed using unpaired Student's *t* tests with Bonferroni adjustment. A *P*-value of <0.05 was considered significant.

## Supporting Information

Figure S1 Recombinant HBHA protein did not increase LDH release in RAW 264.7 cells. RAW 264.7 cells were incubated with the indicated concentrations of HBHA for 48 h. And then LDH release was measured by Cytotoxicity Detection Kit. Positive control was generated by treating cells with 1% Triton X-100 (TX-100) for 1 h prior to the onset of the assay.
(TIF)

Figure S2 DNA fragmentation and LDH release in BMDMs infected with *M. tuberculosis* H37Rv wild type and mutant disrupted in *hbhA*. BMDMs were infected with *M. tuberculosis* wild-type (WT) or mutant (Mu) at indicated MOIs for 24 h. DNA fragmentation (A) and LDH release (B) were measured as described in Figure 1 and Supplemental Figure S1, respectively. *** *P*<0.001 cells infected with *M. tuberculosis* WT *versus* with mutant strain at an MOI of 5. ** *P*<0.01 cells infected with *M. tuberculosis* WT *versus* with mutant strain at an MOI of 10.
(TIF)
