# Supplementary figures and images for: Targeting of Mycobacterium tuberculosis Heparin-Binding Hemagglutinin to Mitochondria in Macrophages
Source: PLoS Pathog. 2011 Dec 8;7(12):e1002435. doi: 10.1371/journal.ppat.1002435 (PMC3234249; doi:10.1371/journal.ppat.1002435)

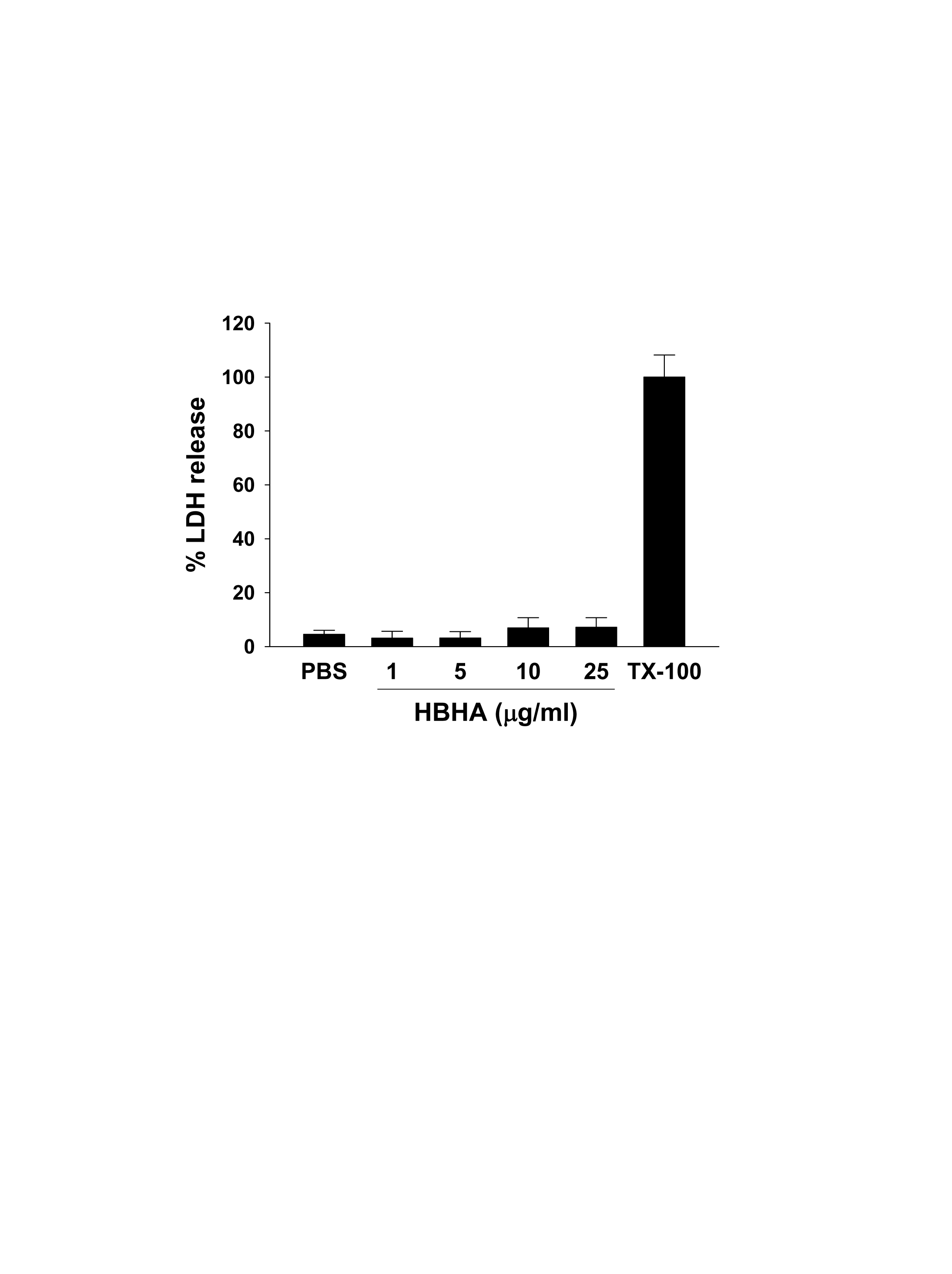

Supplement: Figure S1 — Recombinant HBHA protein did not increase LDH release in RAW 264.7 cells. RAW 264.7 cells were incubated with the indicated concentrations of HBHA for 48 h. And then LDH release was measured by Cytotoxicity Detection Kit. Positive control was generated by treating cells with 1% Triton X-100 (TX-100) for 1 h prior to the onset of the assay. (TIF) [file ppat.1002435.s001.tif]

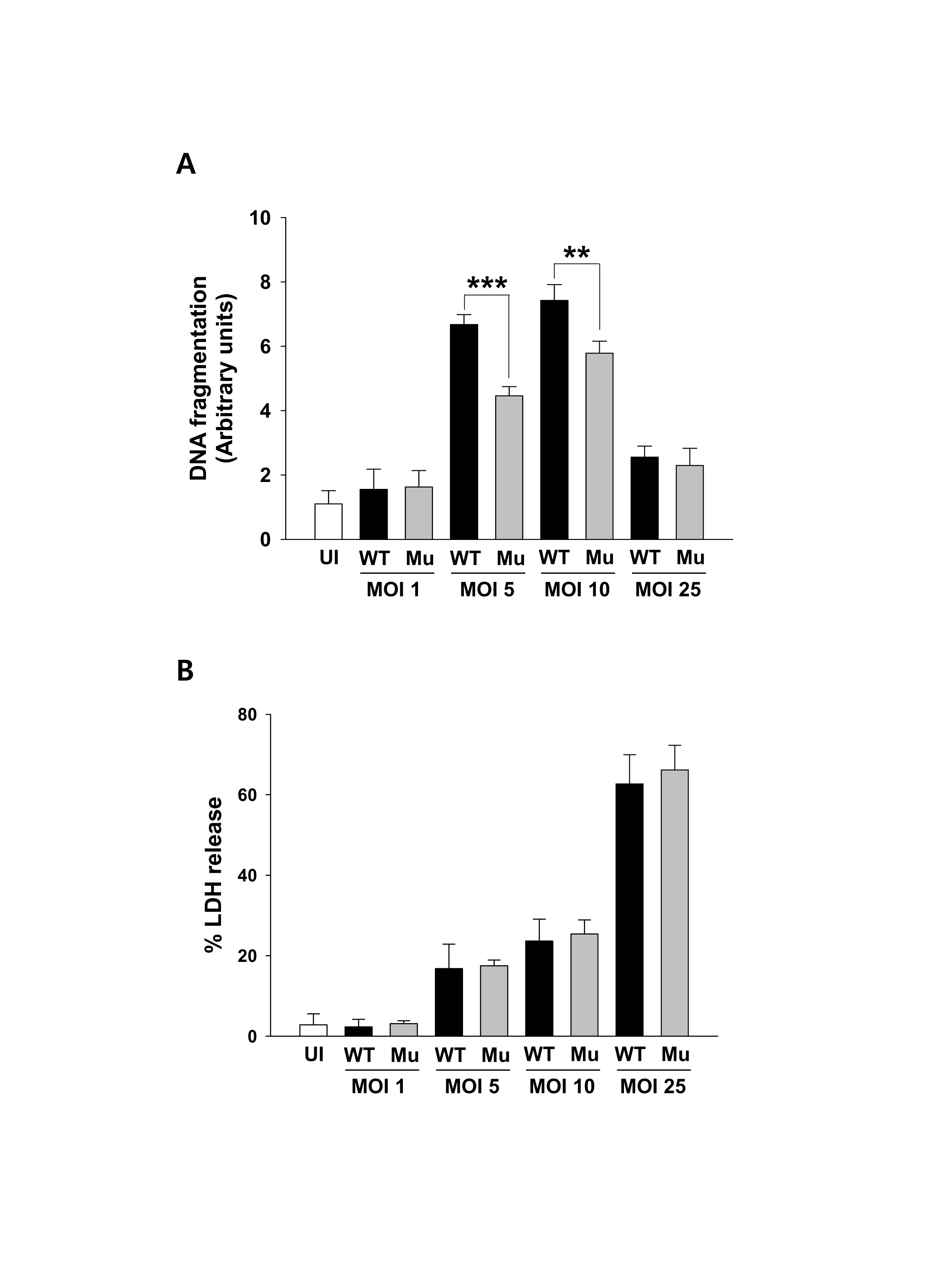

Supplement: Figure S2 — DNA fragmentation and LDH release in BMDMs infected with M. tuberculosis H37Rv wild type and mutant disrupted in hbhA. BMDMs were infected with M. tuberculosis wild-type (WT) or mutant (Mu) at indicated MOIs for 24 h. DNA fragmentation (A) and LDH release (B) were measured as described in Figure 1 and Supplemental Figure S1, respectively. *** P<0.001 cells infected with M. tuberculosis WT versus with mutant strain at an MOI of 5. ** P<0.01 cells infected with M. tuberculosis WT versus with mutant strain at an MOI of 10. (TIF) [file ppat.1002435.s002.tif]
